# Supplementary material for: Normal-Fat vs. High-Fat Diets and Olive Oil vs. CLA-Rich Dairy Fat: A Comparative Study of Their Effects on Atherosclerosis in Male Golden Syrian Hamsters
Source: Metabolites. 2023 Jul 6;13(7):827. doi: 10.3390/metabo13070827 (PMC10383941; doi:10.3390/metabo13070827)
Supplement: Supplementary file 1 [file metabolites-13-00827-s001.zip › Supplementary tables.pdf]

SUPPLEMENTARY TABLE S1. Fatty acids (FA) composition means (mol%) of olive oil (OO) and milk fat (MF), used for preparation of diets for hamsters**.**

	Fat			
**FA**	**OO**	**MF**	**SEM**	**P[1]**
**MCSFA**				
**10:0**	0.00340	11.25	3.248	0.000
**12:0**	0.00512	5.349	1.543	0.000
**14:0**	0.0124	12.80	3.693	0.000
**15:0**	0.00345	1.194	0.3436	0.000
**Sum**	0.0243	30.60	8.827	0.000
**LCSFA**				
**16:0**	12.50	26.59	4.056	0.000
**17:0**	0.0172	0.6699	0.1884	0.000
**18:0**	3.210	11.53	2.403	0.000
**20:0**	0.151	0.2679	0.03599	0.063
**21:0**	0.00382	0.0709	0,01936	0.000
**22:0**	0.0350	0.1015	0.01942	0.011
**24:0**	0.0141	0.0497	0.01034	0.006
**26:0**	0.0195	0.0007	0.00552	0.012
**Sum**	15.95	39.29	6.725	0.000
**BCFA**				
***iso*14:0**	0.000547	0.2293	0.006603	0.000
***iso*15:0**	0.00112	0.4252	0.1224	0.000
***anteiso*15:0**	nd	0.7411	0.2139	0.000
***iso*16:0**	0.00350	0.3781	0.1081	0.000
***iso17*:0**	nd	0.4426	0.2556	0.000
***anteiso*17:0**	0.07740	0.4755	0.2300	0.000
**Sum**	0.08262	2.692	0.7532	0.000
**c-MUFA**				
***cis9*-14:1**	nd	0.2111	0.06163	0.011
***cis9*-16:1**	0.802	0.7850	0.01778	0.725
***cis10*-17:1**	0.0681	0.2284	0.04630	0.001
***cis6*-18:1**	nd	0.0905	0.02613	0.000
***cis9*-18:1**	73.9	18.70	15.94	0.000
***cis11*-18:1**	2.02	0.3748	0.4758	0.001
***cis11*-20:1**	0.0711	0.0343	0.01200	0.113
***cis13*-22:1**	0.00291	0.0002	0.00080	0.018
**Sum**	76.9	20.43	16.29	0.000
**t-MUFA**				
***trans10*-15:1**	nd	nd		
***trans9*-16:1**	nd	0.01631	0.0471	0.000
***trans9*-18:1**	0.0109	0.2834	0.07869	0.000
***trans11*-18:1**	0.00136	2.2312	0.6437	0.000
**Sum**	0.0122	2.531	0.7271	0.000
**PUFA**				
**18:2n-6**	6.067	1.878	1.210	0.001
**18:3n-6**	0.00189	0.07308	0.02059	0.002
**18:3n-3**	0.642	1.133	0.1426	0.001
**20:4 n-6**	0.292	0.07201	0.06664	0.047
**20:5 n-3**	nd	0.1051	0.03051	
**22:6 n-3**	nd	nd		
**Sum PUFAn-6**	6.360	2.023	1.254	0.001
**Sum PUFAn-3**	0.642	1.238	0.1730	0.005
**Sum PUFA**	7.00	3.264	1.081	0.001
**n-6/n-3**	9.923	1.636	2.396	0.001
**CLA**				
***c9,t11*-CLA**	0.00228	1.174	0.3383	0.000
***t10,c12*-CLA**	0.00157	nd	0.00079	0.465
***c9,c11*-CLA**	0.00298	0.00215	0.00124	0.927
***t9,t11*-CLA**	0.00210	0.00540	0.00098	0.011
**Sum**	0.00894	1.182	0.3386	0.000

BCFA: methyl-branched chain FA. CLA: conjugated linoleic acid. c-MUFA: *cis*-monounsaturated FA; LCSFA: long chain saturated FA; MCSFA: medium chain saturated FA; nd: not detected; SFA: saturated FA. SCD-1: Stearoyl-CoA-1 desaturase. t-MUFA: *trans*-monounsaturated FA.

SUPPLEMENTARY TABLE S2. Sequences of primers used in hepatic gene expression analysis

Gene	Exons	Primer Sequences		mT (° C)	Lenght, pb	Accesion No	Ref.
**β-actin**	Exon 4	Forward (3'→ 5')	GTC ATC ACC ATT GGC AAC GA	60	81	NW_004801699	
	Exon 4-5	Reverse (5'→3')	GCC ACA GGA TTC CAT ACC CA				
**PPAR-α**	Exon 6	Forward (3'→ 5')	GAG AAA GCA AAA CTG AAA GCA GAG A	55	180	NW_004801613	Valeille et al.[1]
	Exon 6 - Exon 7	Reverse (5'→3')	GAA GGG CGG GTT ATT GCT G				
**SR-B1**	Exon 6 - Exon 7	Forward (3'→ 5')	AGG CCT GCA GGT CCA TGA AGC	55	129	NW_004801705	
	Exon 7	Reverse (5'→3')	AGA AGC CTT CAT TGG GTG GGT A				
**ACAT**	Exon 10 - Exon 11	Forward (3'→ 5')	GGT GGA ATT ATG TGG CCA AGA	55	101	NW_004801613	
	Exon 11	Reverse (5'→3')	CAT ATT GGC AAA GAC AGG GAC				
**MTP**	Exon 12	Forward (3'→ 5')	ACA TGC TGA CCT TTG TGC GA	60	101	NW_004801723	Lecker et al.[2]
	Exon 13	Reverse (5'→3')	ACG GTC ATA ATT GTG GGC AAC				
**HMG-CoAR**	Exon 14	Forward (3'→ 5')	CGA AGG GTT TGC AGT GAT AAA GGA	55	81	NW_004801607	Huang et al.[3]
	Exon 15	Reverse (5'→3')	GCC ATA GTC ACA TGA AGC TTC TGT A				
**CD36**	Exon 9 - Exon 10	Forward (3'→ 5')	AGG AAT TTG TCC TAT TGG CCA AGT T	55	96	NW_004801709	
	Exon 10 - Exon 11	Reverse (5'→3')	CCG CAG TAC CCG AGA CTT CT				
**LXR**	Exon 3	Forward (3'→ 5')	AAC CCA AGA TGC AAG AGA CC	60	181	NW_004801605	
	Exon 4	Reverse (5'→3')	CTT TTT CCG CTT TTG TGG AC				
**ApoB 100**	Exon 25	Forward (3'→ 5')	ACA CCA GCA GAG ACC ACT TC	65	278	NW_004801692	
	Exon 26	Reverse (5'→3')	AACACTAGCAGACATCTGTGG				
**LDLR**	Exon 10-11	Forward (3'→ 5')	GACCCTGTGCATGGCTTC	55	115	NW_004801687	
	Exon 11	Reverse (5'→3')	GCCACTGGATGTCCTCAGTC				
**ApoA 1**	Exon 2	Forward (3'→ 5')	GGCTGTGCTCTTCCTGAC	62	182	NW_004801622	
	Exon 3 - 4	Reverse (5'→3')	GATTCAGGTTCAGCTGCTTG				

ACAT: acyl-CoA: cholesterol acyltransferase; CD36: cluster of differentiation 36; HMG-CoAR: hydroximethylglutaryl-CoA reductase; LDLR: LDL-receptor; LXR: liver X receptor MTP: microsomal triglyceride transfer protein; PPAR-α: peroxisome proliferator-activated receptor α , SR-B1: HDL scavenger receptor BI.

1. [49] Valeille, K.; Férézou, J.; Amsler, G.; Quignard-Boulangé, A.; Parquet, M.; Gripois, D.; Dorovska-Taran, V.; Martin, J.C. A *cis*-9,*trans*-11-conjugated linoleic acid-rich oil reduces the outcome of atherogenic process in hyperlipidemic hamster. *Am J Physiol Heart Circ Physiol* **2005**, *289*, H652-H659, DOI https://doi.org/10.1152/ajpheart.00130.2005.
2. [60] Lecker, J.L.; Matthan, N.R.; Billheimer, J.T.; Rader, D.J.; Lichtenstein, A.H. Impact of dietary fat type within the context of altered cholesterol homeostasis on cholesterol and lipoprotein metabolism in the F1B hamster. *Metab Clin Exp* **2010**, *59*, 1491-1501, DOI https://doi.org/10.1016/j.metabol.2010.01.014.
3. [61] Huang, H.; Xie, Z.; Yokoyama, W.; Yu, L.; Wang, T.T.Y. Identification of liver CYP51 as a gene responsive to circulating cholesterol in a hamster model. *J Nutr Sci* **2016**, *5*,e16. DOI 10.1017/jns.2016.

SUPPLEMENTARY TABLE S3. Fatty acids (FA) concentration (μmol/L) means (SD) in hamsters plasma.

FA	Hamster groups				
	**OO7[1]**	**OO21[2]**	**MF7[3]**	**MF21[4]**	**P[5]**
**MCSFA**					
**10:0**	26.16 (1.712)	26.06 (1.706)	24.56 (1.772)	30.44 (1.993)	0.108
**12:0**	10.49 (0.8158)[a]	13.48 (1.048)[a]	15.45 (1.350)[a]	46.22 (3.595)[b]	0.000
**14:0**	25.07 (7.644)[a]	31.75 (9.681)[a]	37.16 (19.92)[a]	218.1 (33.24)[b]	0.002
**15:0**	7.428 (0.6782)[a,b]	6.370 (0.5816)[a,b]	5.320 (0.558)[b]	12.36 (1.129)[c]	0.003
**Sum**	69.14 (10.85)[a]	77.65 (13.02)[a]	82.49 (23.60)[a]	307.2 (39.96)[b]	0.002
**LCSFA**					
**16:0**	1634 (107.1)[a,b]	2032 (283.4)[b]	1204 (251.1)[a]	3480 (167.2)[c]	0.002
**17:0**	32.12 (5.460)[a]	35.15 (5.975)[a]	25.74 (5.760)[a]	74.14 (12.60)[b]	0.012
**18:0**	631.4 (103.4)[a]	997.9 (131.2)[b]	371.5 (64.84)[a]	1010.7 (72.62)[b]	0.007
**20:0**	3.423 (0.4626)[a]	5.651 (0.7638)[a]	3.651 (0.610)[a]	9.691 (1.310)[b]	0.005
**21:0**	2.384 (0.4493)	2.644 (0.4981)	1.287 (0.3307)	2.770 (0.5219)	0.094
**22:0**	38.53 (11.62)[a,b]	63.52 (19.15)[b]	10.06 (5.290)[a]	37.18 (11.21)[a,b]	0.061
**24:0**	1.027 (0.5416)	1.254 (0.6613)	0.590 (0.297)	2.035 (1.073)	0.346
**26:0**	4.094 (0.5860)[a]	6.385 (0.9140)[a]	9.039 (1.622)[a]	57.07 (8.169)[b]	0.001
**Sum**	2347 (229.6)[a,b]	3145 (442.6)[b]	1626 (329.8)[a]	4674 (274.7)[b]	0.003
**BCFA**					
***iso*14:0**	1.047 (0.1607)	1.060 (0.1626)	0.9144 (0.1792)	1.147 (0.1760)	0.626
anteiso14:0	1.382 (0.4150)	1.738 (0.5220)	1.119 (0.5841)	2.060 (0.6185)	0.429
***iso*15:0**	4.999 (1.503)[b]	2.161 (0.6497)[a,b]	0.797 (0.417)[a]	2.446 (0.7354)[a,b]	0.043
***anteiso*15:0**	1.430 (0.4573)	3.176 (1.016)	1.811 (1.057)	5.906 (1.889)	0.066
***iso*16:0**	5.267 (0.9478)[a]	5.755 (1.036)[a]	6.242 (1.507)[a]	18.21 (3.277)[b]	0.007
***anteiso*16:0**	0.5306 (0.1555)[a]	nd	nd	0.4754 (0.1393)[a]	0.011
***iso*17:0**	33.22 (8.726)[a]	21.87 (5.745)[a]	31.07 (12.98)[a]	145.6 (38.25)[b]	0.011
***anteiso*17:0**	37.49 (8.155)[a,b]	69.26 (15.07)[b]	6.628 (2.083)[a]	35.05 (7.626)[a,b]	0.013
**Sum**	85.36 (20.52)[a]	105.0 (24.20)[a,b]	48.58 (18.81)[a]	210.9 (52.71)[b]	0.028
**c-MUFA**					
***cis*9-14:1**	29.03 (5.745)[a]	37.71 (7.463)[a]	31.02 (8.525)[a]	109.1 (21.59)[b]	0.008
***cis*9-16:1**	130.7 (26.27)[a]	84.20 (34.41)[a]	141.8 (30.13)[a]	365.6 (63.94)[b]	0.008
***cis*10-17:1**	11.87 (1.426)[a]	6.592 (0.7915)[a]	20.99 (3.036)[a]	60.89 (7.311)[b]	0.001
***cis*6-18:1**	nd	nd	5.886 (2.101)[a]	13.05 (3.096)[b]	0.006
***cis*9-18:1**	2089 (152.4)[a]	3226 (313.6)[b]	1309 (105.7)[a]	3481.8 (506.5)[b]	0.007
***cis*11-18:1**	180.9 (32.12)	173.6 (30.83)	94.25 (22.35)	173.5 (30.81)	0.110
***cis*11-20:1**	12.40 (3.706)	21.22 (6.343)	4.306 (2.229)	16.17 (4.832)	0.078
***cis*13-22:1**	3.661 (0.8913)	4.228 (1.028)	2.078 (0.7703)	3.937 (0.9576)	0.231
**Sum**	2457 (222.6)[a,b]	3554 (394.5)[b,c]	1610 (174.82)[a]	4224 (639.1)[c]	0.010
**t-MUFA**					
***trans*10-15:1**	0.9801 (0.1777)[a]	nd	nd	1.648 (0.2989)[b]	0.002
***trans*9-16:1**	2.271 (0.5338)[a]	1.693 (0.3980)[a]	13.43 (4.730)[b]	2.664 (0.6263)[a]	0.021
***trans*9-18:1**	6.571 (2.233)	9.321 (3.168)	3.732 (2.443)	19.08 (6.485)	0.064
***trans*11-18:1**	4.848 (1.185)[a]	4.797 (1.172)[a]	8.299 (3.100)[a]	42.60 (10.41)[b]	0.006
**Sum**	14.67 (4.130)[a]	15.81 (4.739)[a]	25.46 (10.27)[a]	65.99 (17.82)[b]	0.025
**PUFA**					
**18:2 n-6**	2249 (450.2)[a]	1933 (144.2)[a]	1738 (231.4)[a]	4162 (104.7)[b]	0.003
**18:3 n-6**	81.19 (17.71)[ab]	107.0 (23.34)[b]	21.33 (6.730)[a]	48.71 (10.63)[ab]	0.021
**18:3 n-3**	6.754 (1.801)[a]	10.90 (2.905)[a]	8.585 (3.674)[a]	69.68 (18.58)[b]	0.007
**20:4 n-6**	1146 (168.6)[a]	1320 (194.1)[ab]	699.0 (129.7)[a]	1907 (217.0)[b]	0.012
**20:5 n-3**	6.081 (0.7177)[a]	7.291 (0.8606)[a]	12.03 (1.704)[a]	81.99 (9.677)[b]	0.000
**22:6 n-3**	229.0 (48.70)[a]	482.1 (42.42)[b]	150.6 (45.78)[a]	640.7 (76.14)[b]	0.003
**Sum n-6**	3477 (636.5)[a]	3361 (361.6)[a]	2459 (367.8)[a]	6118 (332.3)[b]	0.005
**Sum n-3**	241.9 (51.21)[a]	500.3 (46.18)[b]	171.2 (51.16)[a]	792.4 (104.4)[c]	0.003
**Sum PUFA**	3719 (687.8)[a]	3860 (407.8)[a]	2630 (419.0)[a]	6910 (436.7)[b]	0.004
**n-6/n-3**	14.42 (0.4219)[b]	6.713 (0.1031)[a]	14.70 (2.244)[b]	7.761 (0.6030)[a]	0.004
**CLA**					
***cis*9, *trans*11-CLA**	4.525 (1.713)[a]	3.965 (1.501)[a]	19.80 (16.12)[a]	191.1 (72.311)[b]	0.018
***trans*10, *cis*12-CLA**	3.125 (0.7917)	3.095 (0.7841)	1.800 (0.7108)	3.004 (0.7612)	0.364
***cis*9,*cis*11-CLA**	7.272 (1.585)[a]	12.37 (2.695)[a]	7.392 (2.328)[a]	26.42 (5.757)[b]	0.015
***trans*9,*trans*11-CLA**	2.664 (0.8641 [a]	3.037 (0.9852)[a]	1.992 (1.194)[a]	14.48 (4.697)[b]	0.020
**Sum**	17.59 (4.953)[a]	22.47 (5.965)[a]	30.98 (20.35)[a]	235.0 (83.53)[b]	0.018
**Total FA**	8711 (1180)[a]	10781 (1293)[a]	6059 (999.4)[a]	16627 (1544)[b]	0.005
SCD1 activity indices					
cis9-16:1/16:0	0.0796 (0.01086)[b]	0.0406(0.01126)[a]	0.1177(0.00048)[b]	0.1047(0.01334)[b]	0.006
cis9-18:1/18:0	3.334 (0.3043)	3.241(0.1119)	3.555(0.3060)	3.4360(0.2543)	0.396
*c9,t11*CLA/*t11*C18:1	0.9175 (0.1290)[a]	0.8126(0.1142)[a]	2.174(1.130)[a]	4.409(0.6198)[b]	0.015

[1]Hamsters fed a diet containing 7% (w) of olive oil. [2]Hamsters fed a diet containing 21% (w) of olive oil. [3]Hamsters fed a diet containing 7% (w) of milk fat. [4]Hamsters fed a diet containing 21% (w) of milk fat. [5] *P*-value < 0.05 was considered statistical significance. [a, b, c] letters indicate significant differences in compared groups. BCFA: methyl-branched chain FA; CLA: conjugated linoleic acid; c-MUFA: cis-monounsaturated FA; LCSFA: long chain saturated FA; MCSFA: medium chain saturated FA; nd: not detected; SFA: saturated FA; SCD-1: Stearoyl-CoA-1 desaturase. t-MUFA: trans-monounsaturated FA.
